# Dose- and time-dependent renoprotection of *Angelica sinensis* in patients with chronic kidney disease: A longitudinal cohort study

**DOI:** 10.3389/fphar.2023.1153583

**Published:** 2023-04-25

**Authors:** Hsiao-Tien Chen, Ben-Hui Yu, Ming-Hsien Yeh, Shih-Kai Hung, Yi-Chun Chen

**Affiliations:** ^1^ Department of Chinese Medicine, Chi Mei Medical Center, Tainan, Taiwan; ^2^ Department of Radiation Oncology, Dalin Tzu Chi Hospital, Buddhist Tzu Chi Medical Foundation, Chiayi, Taiwan; ^3^ Department of Chinese Medicine, Dalin Tzu Chi Hospital, Buddhist Tzu Chi Medical Foundation, Chiayi, Taiwan; ^4^ School of Post-Baccalaureate Chinese Medicine, Tzu Chi University, Hualien, Taiwan; ^5^ School of Medicine, Tzu Chi University, Hualien, Taiwan; ^6^ Division of Nephrology, Department of Internal Medicine, Dalin Tzu Chi Hospital, Buddhist Tzu Chi Medical Foundation, Chiayi, Taiwan

**Keywords:** *Angelica sinensis*, ESRD, CKD, death, dose–response relationship, hyperkalemia risk, renoprotection

## Abstract

**Background:** Based on their anti-oxidative and anti-fibrotic properties, *Angelica sinensis* (Oliv.) Diels roots [Apiaceae; Radix Angelicae sinensis] (Danggui [abbreviated as S in the context]), *Astragalus membranaceus* (Fisch.) Bunge [Fabaceae; *Astragalus membranaceus*] (Huangqi [A]), *Rheum palmatum* L. [Polygonaceae; Rheum palmatum] (Dahuang [R]), and *Salvia miltiorrhiza* Bunge [Lamiaceae; Salvia miltiorrhiza Bunge radix et rhizoma] (Danshen [D]) are potential renoprotective Chinese herbal medicines (CHMs). Renoprotection using ARD alone for the treatment of chronic kidney disease (CKD) has been documented in pre-clinical, clinical, and meta-analysis research; however, only pre-clinical data are available for the use of S alone. Moreover, with an increasing number of CKD patients taking prescribed CHMs, hyperkalemia risk remains unclear.

**Methods:** This study retrospectively analyzed national health insurance claims data in 2001–2017. Propensity score matching was used to analyze renal and survival outcomes and the dose-response effects of S without ARD use in 18,348 new S users, 9,174 new ARD users, and 36,696 non-users. Cox proportional hazard regression was used to investigate adjusted hazard ratios (aHRs) for end-stage renal disease (ESRD) in the presence of competing mortality and death. The additive effect of the S herb in single form to compounds was also analyzed. Additionally, to analyze hyperkalemia risk, an exact match on each covariate was used to include 42,265 new CHM users and non-users, while Poisson regression was used to estimate adjusted incidence rate ratios (aIRRs) of hyperkalemia of prescribed CHMs.

**Results:** S users and ARD users were associated with aHRs of 0.77 (95% confidence interval; 0.69–0.86) and 1.04 (0.91–1.19), respectively, for ESRD and 0.55 (0.53–0.57) and 0.71 (0.67–0.75), respectively, for death. The renal and survival benefits of S use were consistent in several sensitivity analyses. The dose- and time-dependent renoprotection and dose-dependent survival benefits were observed for S use. The top two additive renoprotective collocations of the S herb in compounds were Xue-Fu-Zhu-Yu-Tang and Shen-Tong-Zhu-Yu-Tang, followed by Shu-Jing-Huo-Xue-Tang and Shen-Tong-Zhu-Yu-Tang. Moreover, CHM users were associated with aIRRs of 0.34 (0.31–0.37) for hyperkalemia.

**Conclusion:** This study suggests dose- and time-dependent renoprotection and dose-dependent survival benefits of the S herb in compounds and no increased hyperkalemia risk of the prescribed CHMs in CKD patients.

## 1 Introduction

Chronic inflammation and subsequent oxidative stress, which are at the heart of chronic kidney disease (CKD) pathophysiology, are involved in CKD development and progression (Impellizzeri et al., 2014; Tinti et al., 2021). Regardless of the CKD etiology, they contribute to ultimate renal fibrosis and account for the increased morbidity and cardiovascular and all-cause mortality (Akchurin and Kaskel, 2015). CKD increases the global burden on health systems due to its insidious onset, progressive and debilitating nature, and increasing incidence worldwide (Khan et al., 2022). Renin–angiotensin–aldosterone system inhibitors are the cornerstone therapy for attenuating the progression of CKD to end-stage renal disease (ESRD) because they aim to lowering the intraglomerular pressure-mediated injury, a well-recognized mechanism for structural damage in CKD progression; however, the effect is moderate (Zhong et al., 2013).

Chinese herbal medicine (CHM) interventions have been proposed as a complementary alternative therapy to mitigate CKD progression as they can multi-target inflammation and oxidative stress related to CKD with subsequent benefits in renal fibrosis, as documented in pre-clinical studies, and confer additive or synergistic renoprotective effects, shown in clinical studies when combined with Western medicine (Peng et al., 2005; Zhong et al., 2013; Zhong et al., 2015). *Astragalus membranaceus* (Fisch.) Bunge [Fabaceae; *Astragalus membranaceus*] (Huangqi), *Rheum palmatum* L. [Polygonaceae; Rheum palmatum] (Dahuang), *Salvia miltiorrhiza* Bunge [Lamiaceae; Salvia miltiorrhiza Bunge radix et rhizoma] (Danshen), and *A. sinensis* (Oliv.) Diels roots [Apiaceae; Radix Angelicae sinensis] (Danggui) have been documented to have potential renoprotective effects. *A. membranaceus*, *R. palmatum*, and *S. miltiorrhiza* alone are reported to have renoprotective effects in pre-clinical, clinical, and meta-analysis (Peng et al., 2005; Wang et al., 2012; Zhong et al., 2013; Zhang et al., 2014; Zhong et al., 2015; Ma et al., 2017; Shen et al., 2020) research. *A. sinensis* and *A. membranaceus* are commonly used together to confer renoprotection in pre-clinical and clinical studies. However, the current evidence on whether *A. sinensis* use alone is renoprotective or not is limited to *in vitro* and *in vivo* experimental studies (Yeh et al., 2011; Cheng et al., 2012; Mo et al., 2018).

Two national health insurance (NHI)-based retrospective studies analyzed the data from Taiwan’s NHI program and indicated a substantial percentage of CHM usage in patients with CKD (Lin et al., 2015; Guo et al., 2021). However, whether prescribed CHMs could increase hyperkalemia risk in patients with CKD is unclear because the herbs and CKD are risk factors for hyperkalemia (Palmer, 2004). Therefore, this study aims to investigate the overall and dose–response effects of *A. sinensis* without the use of *A. membranaceus*, *R. palmatum*, or *S. miltiorrhiza* on renal and survival outcomes and the association between prescribed CHMs and hyperkalemia risk in patients with CKD.

## 2 Materials and methods

### 2.1 Data source

CHM services have been covered by a single-payer compulsory Taiwan’s NHI program since 1995, which allows long-term tracking, and reimbursed by the NHI Administration as dosage forms of scientific Chinese medicine powders. All reimbursed CHMs must be produced by pharmaceutical factories possessing the Good Manufacturing Practice certification from Taiwan’s Food and Drug Administration, recommended by Taiwan’s Committee of Chinese Medicine and Pharmacy and prescribed by licensed Chinese medicine physicians. This study retrospectively analyzed a NHI dataset from Taiwan’s 2005 Longitudinal Generation Tracking Database (LGTD 2005), which randomly sampled 2 million beneficiaries from all beneficiaries in the 2015 Taiwan’s NHI program and recorded patient medical information between 2000 and 2017. The database is managed by the Health and Welfare Data Science Center (HWDC) of the Taiwan Ministry of Health and Welfare. The HWDC has validated the representativeness of LGTD 2005, which has been described in detail in previous studies (Chen et al., 2022a; Chen et al., 2022b). The HWDC only provides de-identified data for research. Therefore, patient consent is not required to access LGTD 2005. Patient informed consent and full review were exempted by the Institutional Review Board of the Dalin Tzu Chi Hospital (B10804001). LGTD 2005 adopts ICD-9-CM (before 2016) and ICD-10-CM (after 2016) diagnosis codes to define diseases (Hsieh et al., 2019) and anatomical therapeutic chemical codes to capture drugs.

### 2.2 Study cohort

**FIGURE 1:**
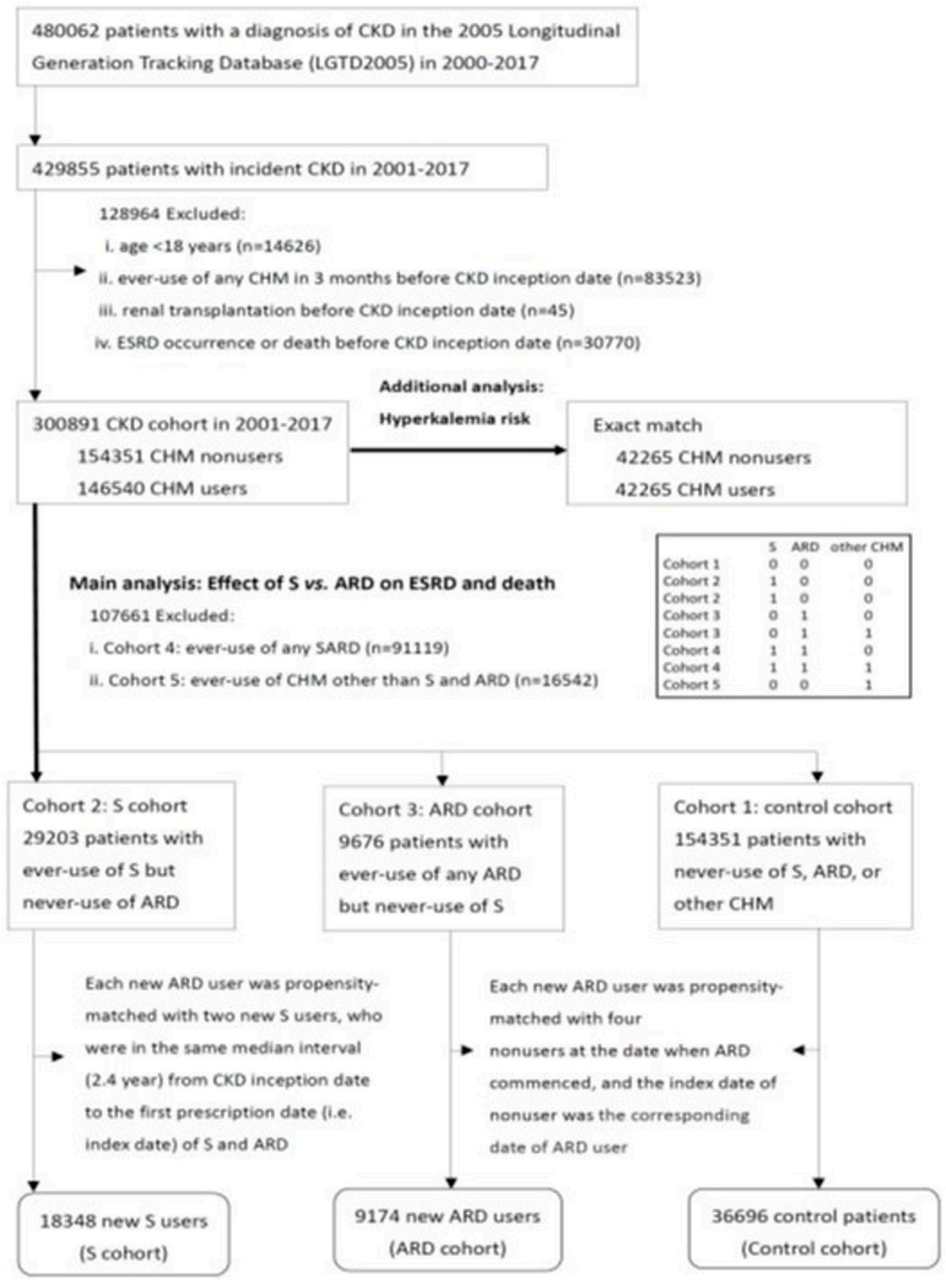
Flow diagram of three study cohorts. Abbreviations: CKD, chronic kidney disease; ESRD, end-stage renal disease; CHM, Chinese herbal medicine; ARD, A: *Astragalus membranaceus* (Fisch.) Bunge, R: *Rheum palmatum* L., and D: Danshen (*Salvia miltiorrhiza* Bunge); S, *Angelica sinensis* (Oliv.) Diels roots.

This study identified 480,062 patients with a claim-based diagnosis of CKD from LGTD 2005 between 2000 and 2017 (Figure 1) and selected 429,855 patients with incident CKD between 2001 and 2017. Overall, 128,964 patients with CKD who were aged <18 years, had taken any CHMs in 3 months, had a renal transplant, experienced ESRD, or died before the CKD inception date were excluded, and an initial CKD cohort of 300,891 patients was obtained.

### 2.3 Study covariates

The covariates included age, sex, baseline comorbidities (including diabetes defined by ICD-9/10-CM codes or anti-diabetic agents, hypertension defined by ICD-9/10-CM codes or anti-hypertensive drugs, coronary heart disease defined by ICD-9/10-CM codes, hyperlipidemia defined by ICD-9/10-CM codes or anti-lipidemic drugs, and chronic liver disease defined by ICD-9/10-CM codes), number of medical visits, Charlson Comorbidity Index in 1 year before the CKD inception date, and two confounding drugs (angiotensin-converting enzyme inhibitors/angiotensin-II receptor antagonists and non-steroid anti-inflammatory drugs).

### 2.4 Study exposure

Based on the prescribed CHMs, 300,891 patients with CKD were divided into CHM users (*n* = 146,540) and non-users (*n* = 154,351) after CKD onset. CHM users were divided into five cohorts during the study period based on the new use of *A. sinensis* (abbreviated as S in the context), new use of *A. membranaceus* (abbreviated as A in the context), *R. palmatum* (abbreviated as R in the context), or *S. miltiorrhiza* (Danshen, abbreviated as D in the context) (Supplementary Table S1) (Chen et al., 2022a); and new use of other CHMs, except for *A. sinensis*, *A. membranaceus*, *R. palmatum*, or *S. miltiorrhiza*. Cohort 1 was the control cohort (no use of any CHM; *n* = 154,351; 51.3%). Cohort 2 comprised patients on the new use of *A. sinensis* but never the use of *A. membranaceus*, *R. palmatum*, or *S. miltiorrhiza*, which was abbreviated as S users in the S cohort (*n* = 29,203; 9.7%). Cohort 3 comprised patients on the new use of any *A. membranaceus*, *R. palmatum*, or *S. miltiorrhiza* but never the use of *A. sinensis*, which was abbreviated as ARD users in the ARD cohort (*n* = 9,676; 3.2%). Cohort 4 (*n* = 91,119; 30.3%) comprised patients on the new use of *A. sinensis*, *A. membranaceus*, *R. palmatum*, or *S. miltiorrhiza*. Cohort 5 (*n* = 16,542; 5.5%) comprised patients on the new use of other CHMs, except for *A. sinensis*, *A. membranaceus*, *R. palmatum*, or *S. miltiorrhiza*. To analyze the effect of S *vs.* ARD use on ESRD and death, cohorts 4 and 5 were excluded from propensity score matching. A new user (exposed to *A. sinensis*, *A. membranaceus*, *R. palmatum*, and *S. miltiorrhiza*) design was used with the follow-up for each ARD user beginning on the date of the first prescription of *A. membranaceus*, *R. palmatum*, or *S. miltiorrhiza* to prevent an immortal bias (Shariff et al., 2008; Chen et al., 2022b). Additionally, each qualified propensity-matched S user and control must have been alive at the time when ARD was commenced. Each new ARD user was matched with two new S users, who were in the same median interval (2.4 years) from the CKD onset date to the first prescription date (index date) of S and ARD. Furthermore, each new ARD user was matched with four non-users at the ARD commencement date, and the index date of the non-user was the corresponding date of the ARD user. The propensity score was calculated using logistic regression built on all study covariates to adjust for baseline differences between ARD users, S users, and non-users. The average standardized mean difference was 0.208 (S *vs.* non-user)/0.137 (ARD *vs.* non-user) and 0.015 (S *vs.* non-user)/0.013 (ARD *vs.* non-user) before and after propensity score matching, respectively. Thus, propensity score matching in this study was well-balanced in the three study cohorts. Finally, 64,218 patients with CKD, comprising 18,348 S users, 9,174 ARD users, and 36,696 non-users, were included in the final analysis.

Moreover, an exact match was used on each covariate among 146,540 CHM users and 154,351 non-users, and 42,265 CHM users and 42,265 non-users were obtained for an additional analysis of hyperkalemia risk of the prescribed CHMs in patients with CKD.

### 2.5 Study outcomes

For the main analysis, all study participants were followed from their index date to the occurrence of ESRD, death, or the end of the study (31 December 2017), whichever occurred first; the latter two were considered as censoring observations. Death before reaching ESRD was considered a competing risk event when estimating the cumulative incidence and risk of ESRD (Hsu et al., 2014a). ESRD was confirmed in the Registry for Catastrophic Illness Patient Database (Hsu et al., 2014b; Chen et al., 2022a), a subset of LGTD 2005. Death was defined as the participant’s withdrawal from the NHI program (Hsu et al., 2014a). For additional analyses, hyperkalemia was defined by the ICD-9/10-CM code, use of potassium-lowering agents, or the procedure code for immediate hemodialysis in the presence of the ICD-9/10-CM code for hyperkalemia.

### 2.6 Statistical analyses

Baseline characteristics between S, ARD, and control cohorts were compared using chi-squared and ANOVA tests for categorical and continuous variables, respectively. The modified Kaplan–Meier and Gray’s methods (Gray, 1988) were used to calculate and compare the cumulative incidences in data with competing risks. The modified Cox proportional hazard model was applied to examine the association of S and ARD use with ESRD and Cox regression for death, with adjustments for all study covariates after confirming the assumption of proportional hazards by plotting the graph of the log (−log(survival)) versus the log of survival time. The CHM prescription day was assessed to address the association between the duration of exposure to S and ARD herbs and the risk of study outcomes among participants having at least a >1 year of follow-up. The duration was measured in cumulative days of use and categorized as 1–30, 31–60, and ≥61 days, with non-use as the reference. The mean dosage of *A. sinensis* prescribed by a licensed CHM practitioner in Taiwan is 1.5 g/d for a single herb and 4–6 g/d for compounds (Chen et al., 2019). The additive effect of *A. sinensis* in one single form to 72 compounds on ESRD risk was also analyzed. Poisson regression was used to estimate the adjusted incident rate ratio of hyperkalemia in association with the prescribed CHM use throughout the study. Recurrent episodes of hyperkalemia were considered separate events if they occurred at least 28 days apart and were considered a long event if they occurred less than 28 days apart (Chen et al., 2022b). In addition, for the main analysis, four sensitivity analyses were conducted to validate the main finding. First, multivariate stratified analyses were conducted on different subgroups. Second, patients with CKD who died or developed ESRD within 30, 60, and 90 days after the index date were excluded to reappraise the risk of study outcomes. Third, S and ARD usage groups were redefined based on the cumulative days of use as >30 days and >60 days. Fourth, three potentially renoprotective drugs sodium-glucose co-transporter 2 inhibitors (Din et al., 2022), glucagon-like peptide-1 agonists (Vitale et al., 2020), and Ketosteril (Wang et al., 2020) were added to the regression model to reappraise the risk of the study outcomes. For additional analyses of hyperkalemia risk, two sensitivity analyses were conducted according to the different exact match models. All data were analyzed using SAS (version 9.4; SAS Institute, Inc., Cary, N.C.). Missing data were not imputed. The statistical significance was set at a two-sided *p*-value less than 0.05.

## 3 Results

### 3.1 Baseline characteristics of the propensity-matched CKD cohort

The average age of the three groups was 54 years, and 52% of them were male (Table 1). No significant difference was observed in the sex, age, comorbidities, Charlson Comorbidity Index, number of medical visits, and confounding drugs among the three groups.

**TABLE 1:** Baseline characteristics of the three study cohorts in CKD patients.

	Propensity-matched CKD patients (*n* = 64,218)			
	S cohort	ARD cohort	Control	
Variable	(*n* = 18,348)	(*n* = 9,174)	(*n* = 36,696)	*p*-value
	N (%)	N (%)	N (%)	
Sex				0.08
Men	9,524 (51.9)	4,850 (52.9)	19,409 (52.9)	
Women	8,824 (48.1)	4,324 (47.1)	17,287 (47.1)	
Age (years)				0.95
18–45	5,885 (32.1)	2,947 (32.1)	11,849 (32.3)	
46–60	5,841 (31.8)	2,915 (31.8)	11,565 (31.5)	
≥61	6,622 (36.1)	3,312 (36.1)	13,282 (36.2)	
Mean (±SD)	53.4 ± 16.6	53.7 ± 16.9	54.7 ± 17.5	
Comorbidities				
Diabetes	5,106 (27.8)	2,550 (27.8)	10,085 (27.5)	0.64
Coronary heart disease	2,138 (11.7)	1,103 (12.0)	4,204 (11.5)	0.30
Hypertension	9,552 (52.1)	4,737 (51.6)	18,952 (51.7)	0.63
Hyperlipidemia	4,551 (24.8)	2,296 (25.0)	9,055 (24.7)	0.77
Chronic liver disease	3,145 (17.1)	1,612 (17.6)	6,344 (17.3)	0.67
No. of medical visits				0.86
1–12	7,358 (40.1)	3,689 (40.2)	14,869 (40.5)	
13–24	5,797 (31.6)	2,898 (31.6)	11,581 (31.6)	
≥25	5,193 (28.3)	2,587 (28.2)	10,246 (27.9)	
Mean (±SD)	19.9 ± 16.7	19.8 ± 16.5	19.4 ± 16.6	
Charlson Comorbidity Index				0.87
≤1	12,152 (66.3)	6,043 (65.8)	24,210 (66.0)	
2	3,344 (18.2)	1,704 (18.6)	6,831 (18.6)	
≥3	2,852 (15.5)	1,427 (15.6)	5,655 (15.4)	
Mean (±SD)	1.12 ± 1.32	1.14 ± 1.35	1.13 ± 1.35	
Confounding drugs				
NSAID	15,171 (82.7)	7,591 (82.7)	30,424 (82.9)	0.79
ACEI/ARB	4,708 (25.7)	2,389 (26.0)	9,422 (25.7)	0.75

Categorical variables are given as numbers (percentage); continuous variables are given as the mean ± standard deviation (SD). Abbreviations: S, *Angelica sinensis* (Oliv.) Diels roots; ARD, A: *Astragalus membranaceus* (Fisch.) Bunge, R: *Rheum palmatum* L., and D: Danshen (*Salvia miltiorrhiza* Bunge); CKD, chronic kidney disease; ACEI/ARB, angiotensin-converting enzyme inhibitor/angiotensin II receptor blocker; NSAID, non-steroid anti-inflammatory drug.

### 3.2 Incidence of ESRD and overall mortality in the three study cohorts

During the follow-up period, 14.4%, 16.4%, and 18.3% of the S, ARD, and control cohorts, respectively, died before developing ESRD (*p*< 0.0001) (Table 2), and 1,666 (2.6%) patients developed ESRD. The 15-year cumulative incidences of ESRD and overall mortality were significantly the lowest in the S cohort [5.1% and 95% confidence interval (CI): 4.5%–5.7%; 38% and 95% CI: 36%–39%], followed by ARD (6.4% and 95% CI: 5.4%–7.5%; 43% and 95% CI: 40%–45%) and control cohorts (7.0% and 95% CI: 6.3%–7.8%; 56% and 95% CI: 54%–58%) (all *p*< 0.0001).

**TABLE 2:** End-stage renal disease occurrence and overall mortality over a 15-year follow-up period.

	S cohort (*n* = 18,348)	ARD cohort (*n* = 9,174)	Control (*n* = 36,696)	*p*-value
ESRD				
Follow-up (years), mean ± SD	5.4 ± 4.2	4.6 ± 3.8	3.3 ± 3.3	
Event number, *n* (%)	450 (2.5)	283 (3.1)	933 (2.5)	
Competing mortality, *n* (%)	2,641 (14.4)	1,502 (16.4)	6,726 (18.3)	<0.0001
Cumulative incidence (%)	5.1 (95% CI: 4.5–5.7)	6.4 (95% CI: 5.4–7.5)	7.0 (95% CI: 6.3–7.8)	<0.0001
Overall mortality				
Follow-up (years), mean ± SD	5.5 ± 4.3	4.7 ± 3.9	3.4 ± 3.3	
Event number, *n* (%)	2,867 (15.6)	1,642 (17.9)	7,231 (19.7)	
Cumulative incidence (%)	38 (95% CI: 36–39)	43 (95% CI: 40–45)	56 (95% CI: 54–58)	<0.0001

Abbreviations: the same as Table 1; SD, standard deviation; CI, confidence interval.

### 3.3 Multivariate-adjusted association of S and ARD use with the study outcomes

Compared with non-use, S use was associated with an adjusted hazard ratio (aHR) of 0.77 (95% CI: 0.69–0.86; *p*< 0.0001) for ESRD and 0.55 (95% CI: 0.53–0.57; *p*< 0.0001) for overall mortality; ARD use was associated with an aHR of 1.04 (95% CI: 0.91–1.19; *p* = 0.59) for ESRD and 0.71 (95% CI: 0.67–0.75; *p*< 0.0001) for overall mortality (Table 3). The benefits of the S herb on renal and survival outcomes remained consistent and statistically significant when adding three potentially renoprotective drugs, sodium-glucose co-transporter 2 inhibitors, glucagon-like peptide-1 agonists, and Ketosteril (Supplementary Table S5).

**TABLE 3:** Adjusted hazard ratios for end-stage renal disease and the overall mortality in the three cohorts.

	ESRD^a^			Overall mortality^b^		
	aHR	95% CI	*p*-value	aHR	95% CI	*p*-value
Control (*n* = 36,696)	1.00	Reference		1.00	Reference	
S cohort (*n* = 18,348)	0.77	0.69–0.86	<0.0001	0.55	0.53–0.57	<0.0001
ARD cohort (*n* = 9,174)	1.04	0.91–1.19	0.59	0.71	0.67–0.75	<0.0001

Abbreviations: the same as Tables 1, 2.

^a^ Adjusted for all covariates (age per year, sex, comorbidities, number of medical visits, Charlson Comorbidity Index, NSAID, and ACEI/ARB) and competing mortality.

^b^ Adjusted for all covariates (age per year, sex, comorbidities, number of medical visits, Charlson Comorbidity Index, NSAID, and ACEI/ARB).

### 3.4 Cumulative exposure duration of S and ARD use and the risk of the study outcomes after at least >1 year of follow-up

Compared with non-use, a graded association was observed between the cumulative exposure duration of S, not ARD, and renoprotection among those exposed to 1–30 (aHR, 0.76; 95% CI: 0.66–0.86; *p*< 0.0001), 31–60 (0.52; 95% CI: 0.31–0.89; *p* = 0.017), and ≥61 (0.46; 95% CI: 0.22–0.98; *p* = 0.045) days (Table 4). However, no graded association was observed between the cumulative exposure duration to S and ARD use and the overall mortality among those exposed for 1–30, 31–60, and ≥61 days.

**TABLE 4:** Cumulative exposure duration of S and ARD use and the risk of the study outcomes of at least >1 year of follow-up.

Cumulative exposure in days	ESRD				Overall mortality			
	N	aHR^a^	95% CI	*p*-value	N	aHR^b^	95% CI	*p*-value
Non-use	25,784	1.00	Reference		26,023	1.00	Reference	
S use								
1–30	14,110	0.76	0.66–0.86	<0.0001	14,190	0.59	0.56–0.63	<0.0001
31–60	889	0.52	0.31–0.89	0.017	891	0.55	0.46–0.65	<0.0001
≥61	504	0.46	0.22–0.98	0.045	506	0.62	0.50–0.77	<0.0001
ARD use								
1–30	6,562	0.83	0.69–0.99	0.034	6,632	0.73	0.68–0.78	<0.0001
31–60	478	1.55	1.01–2.36	0.043	485	0.67	0.54–0.84	0.0005
≥61	383	0.65	0.32–1.30	0.22	392	0.87	0.69–1.09	0.22

Abbreviations: the same as Tables 1–3; ESRD, end-stage renal disease; N, number.

^a^ Adjusted for all covariates (age per year, sex, comorbidities, number of medical visits, Charlson Comorbidity Index, NSAID, and ACEI/ARB) and competing mortality.

^b^ Adjusted for all covariates (age per year, sex, comorbidities, number of medical visits, Charlson Comorbidity Index, NSAID, and ACEI/ARB).

### 3.5 Dose–response relationship of S use with risks of the study outcomes

A dose–response relationship of S use with risks of ESRD and overall mortality was observed when the cumulative S dose was divided into four isometric levels of ≤4.9, 5–9.9, 10–14.9, and ≥ 15 g (Table 5).

**TABLE 5:** Cumulative dose of the exposure to the S herb and the risk of the study outcomes.

Cumulative exposure in doses (grams)^a^	ESRD				Overall mortality			
	Event	aHR^b^	95% CI	*p*-value	Event	aHR^c^	95% CI	*p*-value
Non-use (*n* = 36,696)		1.00	Reference			1.00	Reference	
S use (*n* = 18,348)								
≤4.9 (*n* = 7,892)	225	0.91	0.79–1.05	0.21	1,358	0.64	0.60–0.68	<0.0001
≥5–9.9 (*n* = 4,671)	121	0.81	0.67–0.98	0.029	709	0.55	0.51–0.60	<0.0001
≥10–14.9 (*n* = 1959)	41	0.64	0.47–0.88	0.05	262	0.45	0.40–0.51	<0.0001
≥15 (*n* = 3,826)	63	0.48	0.37–0.62	<0.0001	538	0.43	0.40–0.47	<0.0001

Abbreviations: the same as Tables 1–3; ESRD, end-stage renal disease; N, number.

^a^ The total exposure dose contains the *Angelica sinensis* compound dose ×0.1 and *Angelica sinensis* single herb dose ×1 (Reference 24).

^b^ Adjusted for all covariates (age per year, sex, comorbidities, number of medical visits, Charlson Comorbidity Index, NSAID, and ACEI/ARB) and competing mortality.

^c^ Adjusted for all covariates (age per year, sex, comorbidities, number of medical visits, Charlson Comorbidity Index, NSAID, and ACEI/ARB).

### 3.6 ESRD risk in association with the additive effect of S use in one single form for 72 compounds

The top four orders of additive renoprotection were the use of Xue-Fu-Zhu-Yu-Tang and Shen-Tong-Zhu-Yu-Tang, followed by the use of Shu-Jing-Huo-Xue-Tang and Shen-Tong-Zhu-Yu-Tang, the use of Shen-Tong-Zhu-Yu-Tang, and the use of Shu-Jing-Huo-Xue-Tang (Table 6).

**TABLE 6:** Additive effect of one single form for 72 compounds of S use on end-stage renal disease risk.

Top four orders of additive renoprotection	ESRD event (%)	aHR^a^	95% CI	*p*-value
Xue-Fu-Zhu-Yu-Tang and Shen-Tong-Zhu-Yu-Tang	1 (1.8%)	0.22	0.05–0.91	0.037
Shu-Jing-Huo-Xue-Tang and Shen-Tong-Zhu-Yu-Tang	2 (0.7%)	0.26	0.09–0.76	0.013
Shen-Tong-Zhu-Yu-Tang	5 (0.9%)	0.32	0.12–0.83	0.019
Shu-Jing-Huo-Xue-Tang	46 (2.2%)	0.58	0.41–0.81	0.002

Abbreviations: the same as Tables 2, 3.

^a^ Adjusted for all covariates (age per year, sex, comorbidities, number of medical visits, Charlson Comorbidity Index, NSAID, and ACEI/ARB) and competing mortality.

### 3.7 Hyperkalemia risk

Compared with non-use, the prescribed CHM (adjusted incidence rate ratio, 0.34; 95% CI: 0.31–0.37) usage in patients with CKD was not associated with the increased hyperkalemia risk during the follow-up period (Supplementary Table S2). The results remained consistent despite the different exact match models. S and ARD users also had no increased risk of hyperkalemia during the follow-up period (data not shown).

### 3.8 Sensitivity analyses

**FIGURE 2:**
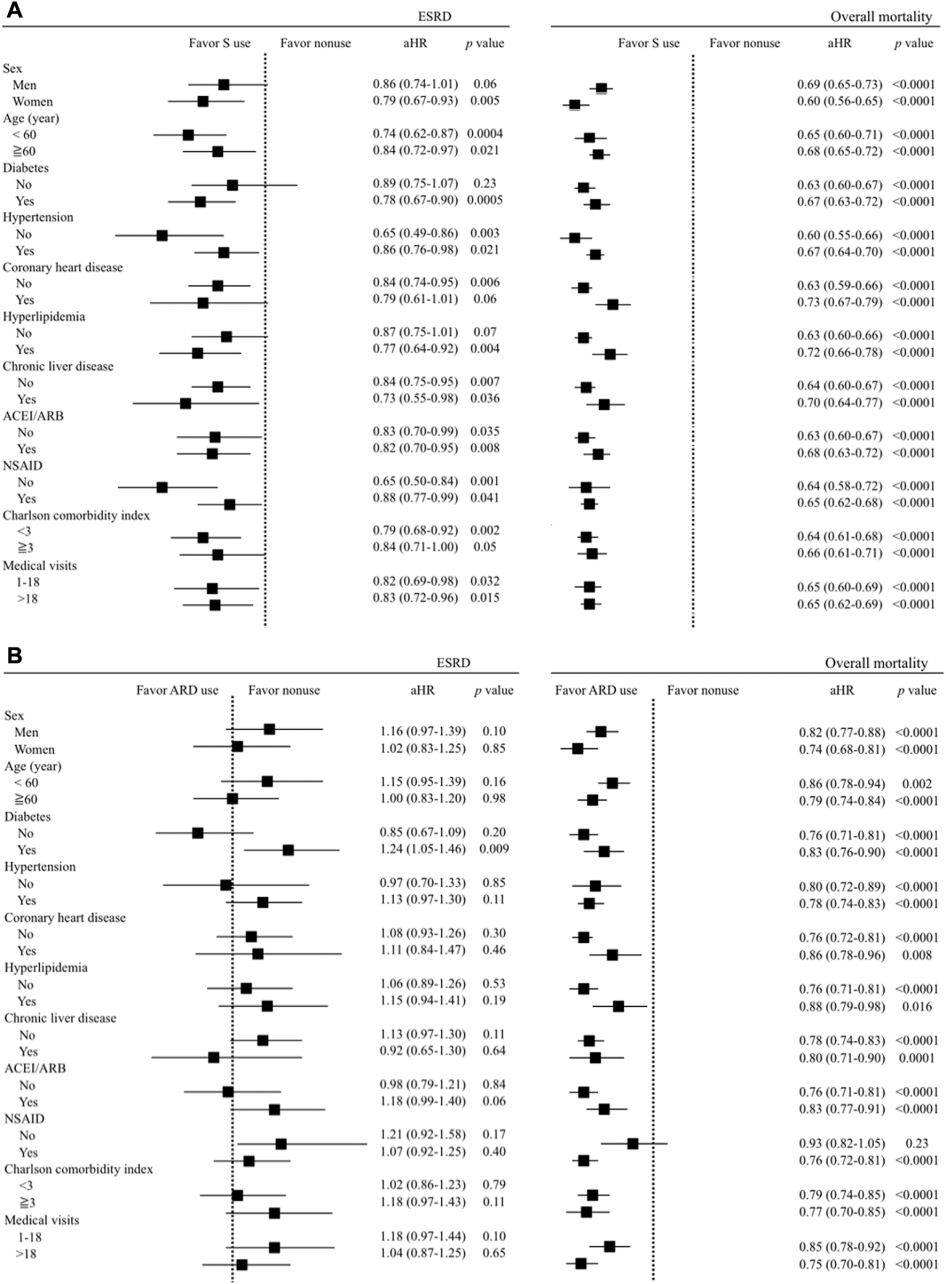
Multivariate stratified analyses for the association between **(A)** S use (*vs.* non-use) and **(B)** ARD use (*vs.* non-use) and the risks of ESRD and overall mortality.

Four sensitivity analyses were conducted to support the reliability of our findings. The association between S use and lower risks of ESRD and overall mortality, and between ARD use and the lower overall mortality remained consistent in three sensitivity analyses, including multivariate stratified analyses (Figure 2), using different definitions of S and ARD use (Supplementary Table S3), and the exclusion of patients with CKD who died or developed ESRD within 30, 60, and 90 days after the index date (Supplementary Table S4).

## 4 Discussion

The key finding of this nationwide cohort study, including 64,218 patients with CKD, is the association between *A. sinensis* in the absence of any *A. membranaceus*, *R. palmatum*, or *S. miltiorrhiza* aid and a 23% reduced ESRD risk. This benefit is dose- and time-dependent, and a higher dose and longer use of *A. sinensis* lower ESRD risks by 52% and 54%, respectively. These findings are reinforced by the experimental model results that suggest biologically plausible mechanisms for renal benefit (Yeh et al., 2011; Cheng et al., 2012; Mo et al., 2018) and are consistent across subgroups and robust in several sensitivity analyses. The top two additive renoprotective collocations are the use of Xue-Fu-Zhu-Yu-Tang and Shen-Tong-Zhu-Yu-Tang and the use of Shu-Jing-Huo-Xue-Tang and Shen-Tong-Zhu-Yu-Tang. In addition, this study adds two novel findings to the existing literature. A dose–response relationship exists between *A. sinensis* and overall mortality. Overall, the prescribed CHMs are not associated with an increased hyperkalemia risk in patients with CKD.

*A. sinensis*, *A. membranaceus*, *R. palmatum*, and *S. miltiorrhiza* are potential renoprotective herbs because of their multi-functional properties of anti-oxidation, anti-inflammation, and anti-fibrosis (Peng et al., 2005; Chao and Lin, 2011; Zhong et al., 2013; Zhong et al., 2015; Xu et al., 2016). The use of *A. membranaceus*, *R. palmatum*, and *S. miltiorrhiza* alone can confer renoprotection, which has been documented in pre-clinical and clinical studies, and meta-analyses (Peng et al., 2005; Wang et al., 2012; Zhong et al., 2013; Zhang et al., 2014; Zhong et al., 2015; Ma et al., 2017; Shen et al., 2020). In contrast, the reported renoprotection of *A. sinensis* alone was only documented in pre-clinical research (Yeh et al., 2011; Cheng et al., 2012; Mo et al., 2018). This study is the first to document the dose- and time-dependent renoprotection of *A. sinensis* without the aid of *A. membranaceus*, *R. palmatum*, and *S. miltiorrhiza* in patients with CKD, which concurred with the previous observations (Yeh et al., 2011; Cheng et al., 2012; Mo et al., 2018). Ferulic acid and ligustilide are considered major bioactive components of *A. sinensis* (Chao and Lin, 2011). In mice with a D-galactose-induced renal injury (Mo et al., 2018), *A. sinensis* treatment, with ligustilide as the major component for 8 weeks, improved the renal function by dose-dependent attenuation of the NF-kB pathway activation and inflammatory cytokine expression and the dose-dependent increase of anti-oxidative enzyme activities and gene expressions, thereby ameliorating renal histological deterioration. In streptozotocin-induced diabetic rats (Yeh et al., 2011; Lv et al., 2018), *A. sinensis* improved the renal function by inhibiting the transforming growth factor (TGF)-β1 expression and collagen IV (Lv et al., 2018) or directly increasing their expression more effectively in a 4-week treatment than 1-week treatment of the renal endogenous bone morphogenetic protein-7 (Yeh et al., 2011). In a mouse model of membranous nephropathy (Cheng et al., 2012), ferulic acid extracted from *A. sinensis* decreased proteinuria dose-dependently and delayed renal progression by reducing oxidative stress. In high glucose-exposed rat mesangial cells (Yeh et al., 2011), the S herb directly scavenged free radicals in a concentration-dependent manner. Furthermore, *A. sinensis* demonstrated a renoprotective potential toward cisplatin-mediated tubulotoxicity (Bunel et al., 2015a) since in addition to being a potent anti-oxidant, ferulic acid has a dose–effect relationship for alleviating cisplatin-induced cell death and apoptosis. This study found that the top two additive renoprotective collocations of *A. sinensis* in compounds were the use of Xue-Fu-Zhu-Yu-Tang and Shen-Tong-Zhu-Yu-Tang and the use of Shu-Jing-Huo-Xue-Tang and Shen-Tong-Zhu-Yu-Tang. These findings are consistent with previous studies (Guo et al., 2013; Wang et al., 2023). The main bioactive compound of Xue-Fu-Zhu-Yu-Tang, Shu-Jing-Huo-Xue-Tang, and Shen-Tong-Zhu-Yu-Tang is Tao Ren [semen of *Prunus persica* (L.) Batsch], which is a potent anti-fibrotic agent that may have a therapeutic potential in patients with fibrotic kidney disease, as it can attenuate renal fibroblast activation and rat renal interstitial fibrosis (Guo et al., 2013). Another common bioactive compound of Xue-Fu-Zhu-Yu-Tang and Shen-Tong-Zhu-Yu-Tang is Hong Hua (*Carthamus tinctorius* L.), which also has therapeutic effects on renal fibrosis (Wang et al., 2023). Given that oxidative stress, inflammation, and fibrosis are three well-documented mechanistic processes implicated in CKD pathogenesis (Khan et al., 2022), *A. sinensis* alone can confer renoprotection as the main effect in clinical practice.

The findings from this study demonstrated a significant survival benefit of *A. sinensis*, *A. membranaceus*, *R. palmatum*, and *S. miltiorrhiza* in patients with CKD, consistent with two prior NHI-based cohort studies. Another randomized clinical trial (Zhang et al., 2020) of 426 patients with acute coronary syndrome and mild-to-moderate renal insufficiency also demonstrated that *A. sinensis* combined with Western medicine improved cardiovascular outcomes in 1 year of follow-up compared with only the use of Western medicine. Cumulative evidence (Tang et al., 2021) has explored vasodilation *in vitro* and anti-hypertensive properties *in vivo* of *A. sinensis*, *A. membranaceus*, *R. palmatum*, and *S. miltiorrhiza*, which are mainly attributed to the regulation of the endothelium-dependent mechanism or vascular smooth muscle cell-mediated mechanism and are effective in treating cardiovascular diseases, such as atherosclerosis, hypertension, and acute coronary syndrome. This study also discovered that *A. sinensis* exhibited a better survival rate than *A. membranaceus*, *R. palmatum*, and *S. miltiorrhiza*, consistent with a prior NHI-based cohort study (Hsieh et al., 2017). This study is the first to document the dose–response effect of *A. sinensis* on overall mortality in patients with CKD. This is consistent with the previous studies that showed high (*vs.* low) doses of *A. sinensis* treatment enhanced cell survival in cisplatin-induced proximal tubule cell toxicity (Bunel et al., 2015a; Bunel et al., 2015b). Furthermore, *A. sinensis* exerted a dose-dependent cardioprotective effect on myocardial ischemia rats by regulating the PI3K/Akt pathway (Cao et al., 2020) and on cardiomyoblast cells by inhibiting angiotensin II-induced apoptosis (Huang et al., 2014). Complementary therapy with *A. sinensis*, *A. membranaceus*, *R. palmatum*, and *S. miltiorrhiza* may offer a considerable potential since patients with CKD are at a high risk for cardiovascular and overall mortality, which is largely driven by cardiovascular deaths (Akchurin and Kaskel, 2015).

CHMs containing aristolochic acid have been withdrawn from the NHI program since November 2003, and Taiwan’s Ministry of Health and Welfare went through a traditional Chinese medicine-enhanced CKD outpatient care program in 2019; however, no consensus has been reached on this issue among nephrologists regarding the suitability of the integrating prescribed CHMs into Western medicine in patients with CKD. Several cohort studies retrospectively analyzed the NHI claims data and documented the renal (Lin et al., 2015; Guo et al., 2021) and survival (Lin et al., 2015; Hsieh et al., 2017; Guo et al., 2021) benefits of the overall prescribed CHMs in patients with CKD, but they did not address if a higher risk of hyperkalemia ensued. Consistent with these two studies, the current study’s CKD population between 2001 and 2017 had 48.7% of prescribed CHM usage after CKD inception, regardless of the indications. Therefore, addressing this concern is crucial. This study is the first to document no increased hyperkalemia risk after a 16-year follow-up in patients with CKD taking prescribed CHMs. This finding was consistent with our previous work (Chen et al., 2022a), documenting no increased hyperkalemia risk in advanced CKD patients taking prescribed *A. sinensis*, *A. membranaceus*, *R. palmatum*, and *S. miltiorrhiza*. Several manufacturing processes, including extraction, decoration, concentration, and granulation, of scientific Chinese medicine powders from raw herb materials may account for the substantially low potassium content of the raw herbs.

This study undertook several methods for minimizing the bias and potential confounding factors, including a relatively large sample size from the universal coverage of a nationwide population and long-term tracking of all study outcomes. First, the enrolled participants were matched with the propensity scores to optimize the comparability among study cohorts. Second, the time when patients received *A. sinensis*, *A. membranaceus*, *R. palmatum*, and *S. miltiorrhiza* was set as the baseline for matching and observation entry to preclude the possibility of an immortal time bias. Third, considering competing mortality prevents an overestimation of non-fatal outcomes in the control cohort (Hsu et al., 2014b).

The study also had several limitations. First, self-paid or non-prescribed CHM is not included in LGTD 2005. Second, the indications of *A. sinensis*, *A. membranaceus*, *R. palmatum*, and *S. miltiorrhiza* could not be determined because of the study’s retrospective design. Thus, a causal association between a drug of interest and the risk of study outcomes cannot be inferred based on an observational study. Furthermore, Taiwan’s Committee of Chinese Medicine and Pharmacy provided *A. sinensis*, *A. membranaceus*, *R. palmatum*, and *S. miltiorrhiza* formulas. However, caution is, thus, recommended before directly applying our results in the West. Third, the adherence to the prescribed CHMs, herb–drug interactions between the prescribed CHM and Western medicine, and pulse-taking diagnoses were unavailable in LGTD 2005. Fourth, LGTD 2005 lacked the exact main etiology of CKD (Hsu et al., 2014a; Chen et al., 2022b), laboratory data, family history, and lifestyle information, which might impact the study outcomes. Fifth, as in any observational study, unmeasured confounders may still exist.

## 5 Conclusion

This nationwide analysis suggests that *A. sinensis* in compounds is associated with dose- and time-dependent renoprotection and dose-dependent survival benefits. Furthermore, prescribed CHMs are not associated with hyperkalemia risk in patients with CKD. The causal relationship mechanisms underlying this association warrant further prospective research.

## Data Availability

The original contributions presented in the study are included in the article/Supplementary Material; further inquiries can be directed to the corresponding author.

## Abbreviations

CKD, chronic kidney disease; ESRD, end-stage renal disease; LGTD 2005, 2005 Longitudinal Generation Tracking Database; NHI, National Health Insurance; S, *Angelica sinensis* (Oliv.) Diels roots; A, *Astragalus membranaceus* (Fisch.) Bunge; R, *Rheum palmatum* L; D, Danshen (*Salvia miltiorrhiza* Bunge).

## References

[B1] Akchurin O. M. Kaskel F. (2015). Update on inflammation in chronic kidney disease. Blood. Purif. 39 (1-3), 84–92. 10.1159/000368940 25662331

[B2] Bunel V. Antoine M. H. Nortier J. Duez P. Stevigny C. (2015a). Nephroprotective effects of ferulic acid, Z-ligustilide and E-ligustilide isolated from Angelica sinensis against cisplatin toxicity *in vitro* . Toxicol. Vitro. 29 (3), 458–467. 10.1016/j.tiv.2014.12.017 25561245

[B3] Bunel V. Antoine M. H. Nortier J. Duez P. Stévigny C. (2015b). Potential nephroprotective effects of the Chinese herb Angelica sinensis against cisplatin tubulotoxicity. Pharm. Biol. 53 (7), 985–994. 10.3109/13880209.2014.951726 25495691

[B4] Cao Y. Liang X. Li C. Chen T. Li Z. Li W. (2020). Experimental study on the effect of aconite and Angelica sinensis on myocardial ischemia rats with yang deficiency and blood stasis. Evid. Based. Complement. Altern. Med. 7027391, 7027391. 10.1155/2020/7027391 PMC7199600 32419818

[B5] Chao W. W. Lin B. F. (2011). Bioactivities of major constituents isolated from Angelica sinensis (Danggui). Chin. Med. 6, 29. 10.1186/1749-8546-6-29 21851645 PMC3170324

[B6] Chen J. Y. Wang Y. H. Hidajah A. C. Li C. Y. (2019). A population-based case-control study on the association of Angelica sinensis exposure with risk of breast cancer. J. Tradit. Complement. Med. 10 (5), 454–459. 10.1016/j.jtcme.2019.10.003 32953561 PMC7484959

[B7] Chen Y. C. Chen H. T. Yeh C. C. Hung S. K. Yu B. H. (2022a). Four prescribed Chinese herbal medicines provide renoprotection and survival benefit without hyperkalemia risk in patients with advanced chronic kidney disease: A nationwide cohort study. Phytomedicine 95, 153873. 10.1016/j.phymed.2021.153873 34896898

[B8] Chen Y. C. Chen Y. C. Chiou W. Y. Yu B. H. (2022b). Impact of acid suppression therapy on renal and survival outcomes in patients with chronic kidney disease: A Taiwanese nationwide cohort study. J. Clin. Med. 11, 5612. 10.3390/jcm11195612 36233478 PMC9570958

[B9] Cheng C. W. Chang W. L. Chang L. C. Wu C. C. Lin Y. F. Chen J. S. (2012). Ferulic acid, an Angelica sinensis-derived polyphenol, slows the progression of membranous nephropathy in a mouse model. Evid. Based. Complement. Altern. Med. 161235, 161235. 10.1155/2012/161235 PMC3403610 22844329

[B10] Din U. A. Salem M. M. Abdulazim D. O. (2022). Sodium-glucose cotransporter 2 inhibitors as the first universal treatment of chronic kidney disease. Nefrol. Engl. Ed. 42, 390–403. 10.1016/j.nefroe.2022.08.001 36460429

[B11] Gray R. J. (1988). A class of K-sample tests for comparing the cumulative incidence of a competing risk. Ann. Stat. 16 (3), 1141–1154.

[B12] Guo J. C. Pan H. C. Yeh B. Y. Lu Y. C. Chen J. L. Yang C. W. (2021). Associations between using Chinese herbal medicine and long-term outcome among pre-dialysis diabetic nephropathy patients: A retrospective population-based cohort study. Front. Pharmacol. 12, 616522. 10.3389/fphar.2021.616522 33679399 PMC7930622

[B13] Guo J. Wu W. Sheng M. Yang S. Tan J. (2013). Amygdalin inhibits renal fibrosis in chronic kidney disease. Mol. Med. Rep. 7, 1453–1457. 10.3892/mmr.2013.1391 23525378

[B14] Hsieh C. F. Chang H. C. Huang S. L. Chen C. L. Chen W. T. Yang C. C. (2017). Prescribed renoprotective Chinese herbal medicines were associated with a lower risk of all-cause and disease-specific mortality among patients with chronic kidney disease: A population-based follow-up study in taiwan. Evid. Based. Complement. Altern. Med. 5632195, 5632195. 10.1155/2017/5632195 PMC5535732 28798802

[B15] Hsieh C. Y. Su C. C. Shao S. C. Sung S. F. Lin S. J. Kao Yang Y. H. (2019). Taiwan's national health insurance research database: Past and future. Clin. Epidemiol. 11, 349–358. 10.2147/CLEP.S196293 31118821 PMC6509937

[B16] Hsu T. W. Liu J. S. Hung S. C. Kuo K. L. Chang Y. K. Chen Y. C. (2014a). Renoprotective effect of renin-angiotensin-aldosterone system blockade in patients with predialysis advanced chronic kidney disease, hypertension, and anemia. JAMA Intern Med. 174, 347–354. 10.1001/jamainternmed.2013.12700 24343093

[B17] Hsu Y. C. Lin J. T. Ho H. J. Kao Y. H. Huang Y. T. Hsiao N. W. (2014b). Antiviral treatment for hepatitis C virus infection is associated with improved renal and cardiovascular outcomes in diabetic patients. Hepatology 59 (4), 1293–1302. 10.1002/hep.26892 24122848

[B18] Huang C. Y. Kuo W. W. Kuo C. H. Tsai F. J. Liu P. Y. Hsieh D. J. (2014). Protective effect of Danggui (Radix Angelicae Sinensis) on angiotensin II-induced apoptosis in H9c2 cardiomyoblast cells. Bmc. Complement. Altern. Med. 14, 358. 10.1186/1472-6882-14-358 25256260 PMC4182826

[B19] Impellizzeri D. Esposito E. Attley J. Cuzzocrea S. (2014). Targeting inflammation: New therapeutic approaches in chronic kidney disease (CKD). Pharmacol. Res. 81, 91–102. 10.1016/j.phrs.2014.02.007 24602801

[B20] Khan M. A. Kassianos A. J. Hoy W. E. Alam A. K. Healy H. G. Gobe G. C. (2022). Promoting plant-based therapies for chronic kidney disease. J. Evid. Based. Integr. Med. 27, 2515690X221079688. 10.1177/2515690X221079688 PMC8902019 35243916

[B21] Lin M. Y. Chiu Y. W. Chang J. S. Lin H. L. Lee C. T. Chiu G. F. (2015). Association of prescribed Chinese herbal medicine use with risk of end-stage renal disease in patients with chronic kidney disease. Kidney. Int. 88 (6), 1365–1373. 10.1038/ki.2015.226 26244923

[B22] Lv W. Booz G. W. Fan F. Wang Y. Roman R. J. (2018). Oxidative stress and renal fibrosis: Recent insights for the development of novel therapeutic strategies. Front. Physiol. 9, 105. 10.3389/fphys.2018.00105 29503620 PMC5820314

[B23] Ma Z. G. Xia H. Q. Cui S. L. Yu J. (2017). Attenuation of renal ischemic reperfusion injury by salvianolic acid B via suppressing oxidative stress and inflammation through PI3K/Akt signaling pathway. Braz. J. Med. Biol. Res. 50 (6), e5954. 10.1590/1414-431X20175954 28513773 PMC5479385

[B24] Mo Z. Z. Lin Z. X. Su Z. R. Zheng L. Li H. L. Xie J. H. (2018). Angelica sinensis supercritical fluid CO(2) extract attenuates D-galactose-induced liver and kidney impairment in mice by suppressing oxidative stress and inflammation. J. Med. Food. 21 (9), 887–898. 10.1089/jmf.2017.4061 30109956

[B25] Palmer B. F. (2004). Managing hyperkalemia caused by inhibitors of the renin-angiotensin-aldosterone system. *N. Engl. J. Med*. 351 (6), 585–592. 10.1056/NEJMra035279 15295051

[B26] Peng A. Gu Y. Lin S. Y. (2005). Herbal treatment for renal diseases. Ann. Acad. Med. Singap. 34 (1), 44–51. 15726219

[B27] Shariff S. Z. Cuerden M. S. Jain A. K. Garg A. X. (2008). The secret of immortal time bias in epidemiologic studies. J. Am. Soc. Nephrol. 19 (5), 841–843. 10.1681/ASN.2007121354 18322159

[B28] Shen Y. Wang S. Liu Y. Ge L. Xia L. Zhang X. (2020). The effects of salvianolate combined with western medicine on diabetic nephropathy: A systematic review and meta-analysis. Front. Pharmacol. 11, 851. 10.3389/fphar.2020.00851 32595500 PMC7304251

[B29] Tang F. Yan H. L. Wang L. X. Xu J. F. Peng C. Ao H. (2021). Review of natural resources with vasodilation: Traditional medicinal plants, natural products, and their mechanism and clinical efficacy. Front. Pharmacol. 12, 627458. 10.3389/fphar.2021.627458 33867985 PMC8048554

[B30] Tinti F. Lai S. Noce A. Rotondi S. Marrone G. Mazzaferro S. (2021). Chronic kidney disease as a systemic inflammatory syndrome: Update on mechanisms involved and potential treatment. Life (Basel) 11 (5), 419. 10.3390/life11050419 34063052 PMC8147921

[B31] Vitale M. Haxhi J. Cirrito T. Pugliese G. (2020). Renal protection with glucagon-like peptide-1 receptor agonists. Curr. Opin. Pharmacol. 54, 91–101. 10.1016/j.coph.2020.08.018 33027748

[B32] Wang H. Song H. Yue J. Li J. Hou Y. B. Deng J. L. (2012). Rheum officinale (a traditional Chinese medicine) for chronic kidney disease. *Cochrane. Database. Syst. Rev*. 7, CD008000. 10.1002/14651858.CD008000.pub2 PMC11089517 22786510

[B33] Wang J. Li X. Chang H. Si N. (2023). Network pharmacology and bioinformatics study on the treatment of renal fibrosis with persicae semen-carthami flos drug pair. Med. Baltim. 102, e32946. 10.1097/MD.0000000000032946 PMC11309690 36827014

[B34] Wang Y. C. Juan S. H. Chou C. L. Hsieh T. C. Wu J. L. Fang T. C. (2020). Long-term effects of ketoanalogues on mortality and renal outcomes in advanced chronic kidney disease patients receiving a low-protein diet. Nutrients 12, 2708. 10.3390/nu12092708 32899821 PMC7551296

[B35] Xu L. Shen P. Bi Y. Chen J. Xiao Z. Zhang X. (2016). Danshen injection ameliorates STZ-induced diabetic nephropathy in association with suppression of oxidative stress, pro-inflammatory factors and fibrosis. Int. Immunopharmacol. 38, 385–394. 10.1016/j.intimp.2016.06.024 27355131

[B36] Yeh C. H. Chang C. K. Cheng K. C. Li Y. X. Zhang Y. W. Cheng J. T. (2011). Role of bone morphogenetic proteins-7 (BMP-7) in the renal improvement effect of DangGui (Angelica sinensis) in type-1 diabetic rats. Evid. Based. Complement. Altern. Med. 796723, 796723. 10.1093/ecam/nep167 PMC3163074 21876712

[B37] Zhang D. W. Wang S. L. Wang P. L. Du J. P. Gao Z. Y. Wang C. L. (2020). The efficacy of Chinese herbal medicines on acute coronary syndrome with renal insufficiency after percutaneous coronary intervention. J. Ethnopharmacol. 248, 112354. 10.1016/j.jep.2019.112354 31689480

[B38] Zhang H. W. Lin Z. X. Xu C. Leung C. Chan L. S. (2014). Astragalus (a traditional Chinese medicine) for treating chronic kidney disease. Cochrane. Database. Syst. Rev. 10, CD008369. 10.1002/14651858.CD008369.pub2 PMC10589061 25335553

[B39] Zhong Y. Deng Y. Chen Y. Chuang P. Y. He J. C. (2013). Therapeutic use of traditional Chinese herbal medications for chronic kidney diseases. Kidney. Int. 84 (6), 1108–1118. 10.1038/ki.2013.276 23868014 PMC3812398

[B40] Zhong Y. Menon M. C. Deng Y. Chen Y. He J. C. (2015). Recent advances in traditional Chinese medicine for kidney disease. *Am. J. Kidney. Dis*. 66 (3), 513–522. 10.1053/j.ajkd.2015.04.013 26015275

