# Supplementary material for: Dose- and time-dependent renoprotection of Angelica sinensis in patients with chronic kidney disease: A longitudinal cohort study
Source: Front Pharmacol. 2023 Apr 25;14:1153583. doi: 10.3389/fphar.2023.1153583 (PMC10166798; doi:10.3389/fphar.2023.1153583)
Supplement: Supplementary file 1 [file DataSheet1.doc]

| **Supplementary Table 1.** Prescribed Chinese herbal medicines (CHMs) containing SARD covered by Taiwan's National Health Insurance | |
| --- | --- |
| **1. CHMs containing *Angelica sinensis* (Oliv.) Diels roots [Apiaceae; Radix Angelicae sinensis] (Danggui) (abbreviated as S in the context)** | |
| **Single herb** | **Compounds** |
| Dang Gui/Angelica sinensis (Oliv.) Diels  Dang Gui Wei/Angelicae Sinensis Extremitas Radicis | Siwu Tang; BuzhongYiqi Tang; Guipi Tang; Yang Xin Tang; Ginseng Yangrong Tang; BaiheGujin Tang; QinjiaoBiejia Tang; Ba Xian Tang; Ba Zhen Tang; ShiquanDabu Tang; Wuji San; Jia Wei Xiao Yao San; Xiao Yao San; Su Zi Jiang Qi Tang; Shu Jing HuoXue Tang; Xue Fu Ju Yu Tang; Bu Yang Huan Wu Tang; Tao Hong Si Wu Tang; Xiao Feng San; San Bi Tang; DwuHwoJih Sheng Tang; Qing Shu Yi Qi Tang; Wu Lin San; LongdanXiegan Tang; Qingwei San; JinjieLianqiao Tang; ZiyinJianghuo Tang; DangguiLonghui Wan; Qingfei Tang; DangguiLiuhuang Tang; SanzhongKuijian Tang; Tiaojing Wan; FangfengTongsheng San; Fu Yuan HuoXue Tang; Shaoyao Tang; Dang GuiNian Tong Tang; Dang Gui Sini Tang; Yi Yi Ren Tang; Runchang Tang; Wenjing Tang; BaweiDaixia Fang; Qionggui Jiao Ai Tang; DangguiShaoyao San; Shenghua Tang; GulouZhishi Tang; Bu Yin Tang; DangguiBuxie Tang; QibaoMeiran Dan; DangguiYinzi; ChaihuQinggan Tang; Tori Xaiodu Yin; Wen Qing Yin; Yigan San; Yiguan Jian; Da Fang Feng Tang; Da QinjiaoTang; XiaojiYinzi; TianwangBuxin Dan; Shao FuhJwuIu Tang; Yougui Wan; Shen Tong Zhu Yu Tang; XiganMingmu San; Ume Wan; ZhenrenHuoming Yin; Liang Xue Di Huang Tang; Ching Shang JiuanTong Tang; ZishenMingmu Wan; ZishenTonger Tang; Danggui San; Guoqi Yin; GexiaZhuyu Tang; Shuangjie San; Zheng Gu ZiJin Dan; Shao Yao Tang; Juan Bi Tang; San Bi Tang |
| **2. CHMs containing *Astragalus membranaceus* (Fisch.) Bunge [Fabaceae; Astragalus membranaceus] (Huangqi) (abbreviated as A in the context)** | |
| **Single herb** | **Compounds** |
| Huang Qi/ Astragalus membranaceus (Fisch.) Bunge | BuzhongYiqi Tang; Guipi Tang; Yang Xin Tang; Ginseng Yangrong Tang;  YiqiCongming Tang; ShiquanDabu Tang; HuangqiWuwu Tang; Bu Yang Huan Wu Tang; Juan Bi Tang; Qing Shu Yi Qi Tang; QingxinLianzi Yin; DangguiLiuhuang Tang; Sheng Yu Tang; Huang Qi Jianzhong Tang; Banxia Ma Baizhu Tang; Yupingfeng San; Yu Quan Wan; Jiegeng Tang; DangguiBuxie Tang; Zaizao San; BaochanWuyou Fang; DangguiYinzi; Tori Xaiodu Yin; ShiliuweiLiuqi Yin; QianjinNeto San; ShengyangYiwei Tang; Fang Ji Huang Qi Tang; San Bi Tang; Da Fang Feng Tang |
| **3. CHMs containing *Rheum palmatum* L. [Polygonaceae; Rheum palmatum] (Dahuang) (abbreviated as R in the context)** | |
| **Single herb** | **Compounds** |
| Da Huang/ Rheum rhabarbarum L. | Da Cheng Qi Tang; Di Dang Tang; Jenq Gu TzyJin Dan; Ba Zheng San; Liang Ge San; Hsiao Cheng Qi Tang; Tiao Wei Cheng Qi Tang; Tao Ren Cheng Qi Tang; Da Chai Hu Tang; FangfengTongsheng San; Da Huang Mu Dan Tang; Fu Yuan HuoXue Tang; Shaoyao Tang; Runchang Tang; Runchang Wan; XiangshengPodiWan; SanhuangXiexin Tang; QingyanLige Tang; Yi Zi Tang; BaweiDiaxia Tang; HuanglianShangqing Wan; MuxiangBinlang Wan; Maziren Wan |
| **4. CHMs containing *Salvia miltiorrhiza* Bunge [Lamiaceae; Salvia miltiorrhiza Bunge radix et rhizoma]** **(Danshen) (abbreviated as D in the context)** | |
| **Single herb** | **Compound** |
| Dan Shen/Salvia miltiorrhiza Bunge | Compound Danshen Tablets |

Note: It shows as herbal product name or single herb name/Latin name

| **Supplementary Table 2.** Hyperkalemia occurrence during follow-up | | | |
| --- | --- | --- | --- |
|  |  | CHM users | Nonusers |
| **Exact match model 1^#^** |  | n=42265 | n=42265 |
| Inpatient and outpatient events |  | 657 | 598 |
| Person-years observed |  | 417298 | 263073 |
| Incidence rate per 100 person-years |  | 0.16 | 0.23 |
| Adjusted incidence rate ratio^*^ (95% CI) |  | 0.34 (0.31-0.37) | 1 (reference) |
| **Sensitivity analysis** |  |  |  |
| Exact match model 2: exact match model 1+index year |  | n=13075 | n=13075 |
| Adjusted incidence rate ratio^*^ (95% CI) |  | 0.92 (0.74-1.15) | 1 (reference) |
| Exact match model 3: exact match model 1 with age, CCI, number of visits in category rather than in integer |  | n=106,047 | n=106,047 |
| Adjusted incidence rate ratio^*^ (95% CI) |  | 0.26 (0.25-0.27) | 1 (reference) |
| Abbreviations: the same as Tables 1-3.  ^#^Individuals were paired up only when their each and every covariate was exactly the same. Age, Charlson comorbidity index (CCI), and number of clinical visits matched to integers rather than categories.  ^*^Adjusted for all covariates (age per year, sex, comorbidities, number of medical visits, CCI, NSAID, and ACEI/ARB). | | | |

| **Supplementary Table 3.** Sensitivity test 1: Study outcomes by definitions of S and ARD use | | | | | | | |
| --- | --- | --- | --- | --- | --- | --- | --- |
| Definition of herbal use | ESRD^*^ | | |  | Overall mortality^#^ | | |
|  | aHR | 95% CI | *p*-value |  | aHR | 95% CI | *p*-value |
| Over 30 days as use |  |  |  |  |  |  |  |
| Nonuse (n=25784) | 1.00 | reference |  |  | 1.00 | reference |  |
| S use (n=1393) | 0.50 | 0.32-0.78 | 0.002 |  | 0.58 | 0.50-0.67 | <0.0001 |
| ARD use (n=861) | 1.11 | 0.76-1.61 | 0.60 |  | 0.76 | 0.65-0.89 | 0.0007 |
| Over 60 days as use |  |  |  |  |  |  |  |
| Nonuse (n=25784) | 1.00 | reference |  |  | 1.00 | reference |  |
| S use (n=504) | 0.47 | 0.22-0.99 | 0.047 |  | 0.63 | 0.50-0.78 | <0.0001 |
| ARD use (n=383) | 0.65 | 0.33-1.31 | 0.23 |  | 0.87 | 0.70-1.10 | 0.24 |
| Abbreviations: the same as Tables 1-3; ESRD, end-stage renal disease.  ^*^Adjusted for all covariates (age per year, sex, comorbidities, number of medical visits, Charlson comorbidity index, NSAID, and ACEI/ARB) and competing mortality.  ^#^Adjusted for all covariates (age per year, sex, comorbidities, number of medical visits, Charlson comorbidity index, NSAID, and ACEI/ARB). | | | | | | | |

| **Supplementary Table 4.** Sensitivity test 2: Risks of study outcomes in CKD patients excluding dying or developing ESRD within 30, 60, and 90 days after the index date | | | | | | | | |
| --- | --- | --- | --- | --- | --- | --- | --- | --- |
|  |  |  | ESRD | |  |  | Overall mortality | |
|  |  | N | Crude HR (95% CI) | Adjusted HR^*^ (95% CI) |  | N | Crude HR (95% CI) | Adjusted HR^#^ (95% CI) |
| Follow-up  >30 days | Control | 35436 | 1.00 (Reference) | 1.00 (Reference) |  | 35475 | 1.00 (Reference) | 1.00 (Reference) |
|  | S | 18083 | 0.72 (0.65-0.81) | 0.78 (0.70-0.88) |  | 18086 | 0.50 (0.48-0.53) | 0.56 (0.53-0.58) |
|  | ARD | 8983 | 0.97 (0.84-1.11) | 1.02 (0.89-1.17) |  | 8997 | 0.67 (0.63-0.71) | 0.71 (0.67-0.75) |
| Follow-up  >60 days | Control | 34275 | 1.00 (Reference) | 1.00 (Reference) |  | 34332 | 1.00 (Reference) | 1.00 (Reference) |
|  | S | 17824 | 0.71 (0.64-0.80) | 0.77 (0.69-0.87) |  | 17837 | 0.51 (0.49-0.53) | 0.56 (0.54-0.59) |
|  | ARD | 8805 | 0.95 (0.83-1.09) | 1.00 (0.87-1.15) |  | 8827 | 0.67 (0.63-0.70) | 0.71 (0.67-0.75) |
| Follow-up  >90 days | Control | 33211 | 1.00 (Reference) | 1.00 (Reference) |  | 33292 | 1.00 (Reference) | 1.00 (Reference) |
|  | S | 17535 | 0.72 (0.64-0.80) | 0.78 (0.69-0.87) |  | 17553 | 0.51 (0.48-0.53) | 0.56 (0.54-0.59) |
|  | ARD | 8658 | 0.92 (0.80-1.06) | 0.96 (0.84-1.11) |  | 8692 | 0.66 (0.63-0.70) | 0.71 (0.67-0.75) |
| Abbreviations: the same as Tables 1-3; ESRD, end-stage renal disease.  ^*^Adjusted for all covariates (age per year, sex, comorbidities, number of medical visits, Charlson comorbidity index, NSAID, and ACEI/ARB) and competing mortality.  ^#^Adjusted for all covariates (age per year, sex, comorbidities, number of medical visits, Charlson comorbidity index, NSAID, and ACEI/ARB). | | | | | | | | |

| **Supplementary Table 5.** Sensitivity test 3: Adjusted hazard ratios (aHRs) for end-stage renal disease (ESRD) and overall mortality in three cohorts when adding three potentially renoprotective drugs (sodium-glucose co-transporter-2 inhibitors, [glucagon-like peptide-1 agonists](https://www.whocc.no/atc_ddd_index/?code=A10BJ&showdescription=no), and Ketosteril) | | | | | | | |
| --- | --- | --- | --- | --- | --- | --- | --- |
|  | ESRD^*^ | | |  | Overall mortality^#^ | | |
|  | aHR | 95% CI | *p*-value |  | aHR | 95% CI | *p*-value |
| Control (n=36696) | 1.00 | Reference |  |  | 1.00 | Reference |  |
| S cohort (n=18348) | 0.77 | 0.69-0.86 | <0.0001 |  | 0.55 | 0.53-0.57 | <0.0001 |
| ARD cohort (n=9174) | 1.04 | 0.90-1.18 | 0.61 |  | 0.71 | 0.67-0.75 | <0.0001 |
| Abbreviations: the same as Tables 1-2.  ^*^Adjusted for all covariates (age per year, sex, comorbidities, number of medical visits, Charlson comorbidity index, NSAID, ACEI/ARB, sodium-glucose co-transporter-2 inhibitors, [glucagon-like peptide-1 agonists](https://www.whocc.no/atc_ddd_index/?code=A10BJ&showdescription=no), and Ketosteril) and competing mortality.  ^#^Adjusted for all covariates (age per year, sex, comorbidities, number of medical visits, Charlson comorbidity index, NSAID, ACEI/ARB, sodium-glucose co-transporter-2 inhibitors, [glucagon-like peptide-1 agonists](https://www.whocc.no/atc_ddd_index/?code=A10BJ&showdescription=no), and Ketosteril). | | | | | | | |
